# Chronic kidney disease after liver, cardiac, lung, heart–lung, and hematopoietic stem cell transplant

**DOI:** 10.1007/s00467-008-0785-2

**Published:** 2008-06-01

**Authors:** Sangeeta Hingorani

**Affiliations:** Pediatrics–University of Washington, 4800 Sandpoint Way NE M1–5, Seattle, WA 98015 USA

**Keywords:** Chronic kidney disease, Liver transplant, Cardiac transplant, Hematopoietic stem cell transplant, Calcineurin inhibitors, Risk factors, Epidemiology, Lung transplant

## Abstract

Patient survival after cardiac, liver, and hematopoietic stem cell transplant (HSCT) is improving; however, this survival is limited by substantial pretransplant and treatment-related toxicities. A major cause of morbidity and mortality after transplant is chronic kidney disease (CKD). Although the majority of CKD after transplant is attributed to the use of calcineurin inhibitors, various other conditions such as thrombotic microangiopathy, nephrotic syndrome, and focal segmental glomerulosclerosis have been described. Though the immunosuppression used for each of the transplant types, cardiac, liver and HSCT is similar, the risk factors for developing CKD and the CKD severity described in patients after transplant vary. As the indications for transplant and the long-term survival improves for these children, so will the burden of CKD. Nephrologists should be involved early in the pretransplant workup of these patients. Transplant physicians and nephrologists will need to work together to identify those patients at risk of developing CKD early to prevent its development and progression to end-stage renal disease.

## Introduction

Chronic kidney disease (CKD) is a frequent and increasingly recognized complication of solid-organ and hematopoietic stem cell transplantation (HSCT), increasing the complexity of patient management and impacting survival. The incidence of CKD after cardiac, liver, and HSCT varies from 7–86% in pediatric patients, partly due to the lack of a uniform definition of CKD after transplant (Table 1). Up to 16% of transplant survivors will develop end-stage renal disease (ESRD) [1–19].

**Table 1:** Percentage of patients with chronic kidney disease (CKD) and end-stage renal disease (ESRD) based on transplant type

Transplant type	CKD	ESRD	Reference
Liver	28–86%	0–8%	[1–5]
Cardiac	7–54%	2%	[6–10]
Heart–lung	34%	7–16%	[11–13]
Hematopoietic stem cell transplantation	18–42%	5–8%	[14–19]

This review focuses on the epidemiology, risk factors, and outcomes of children who develop CKD after liver, cardiac, lung, heart–lung, and stem cell transplant.

### Liver transplant

#### Epidemiology

The majority of studies of renal disease after liver transplantation has been done in adults, and the cumulative incidence of CKD varies from 5% to 30% over 1–5 years after transplant [20]. In a retrospective study of 117 pediatric liver transplant patients who survived 3 years after transplant (median 7.6, range 3–14.6 years), the prevalence of CKD, defined as a glomerular filtration rate (GFR) <70 ml/min per 1.73 m^2^ at last follow-up was 32% [1]. In a 10-year follow-up study of 12 children undergoing a liver transplant in France, mild to moderate CKD (mild = GFR 60–80 ml/min per 1.73 m^2^; moderate = GFR 20–60 ml/min per 1.73 m^2^) developed in six of seven patients. Early deterioration of renal function is usually seen within the first year following transplant, followed by a period of stabilization, then with progression on long-term follow-up [2, 3]. In contrast, in a study of 50 pediatric liver transplant patients in Poland, CKD stages 2 and 3 as defined by the National Kidney Foundation Kidney Disease Outcomes Quality Initiative (NKF/KDOQI) (Table 2) developed at 1 year after transplant and remained stable after 3 years of follow-up [4]. In this study, GFR was calculated using both diethylenetriamine pentaacetic acid (DTPA) plasma clearance and estimated using the Schwartz formula. Little correlation was found between the two methods with the Schwartz formula, consistently overestimating GFR [4]. Therefore, the prevalence of CKD is likely underestimated in this patient population if estimated measures of GFR or serum creatinine are used to define CKD.

**Table 2:** National Kidney Foundation Kidney Disease Outcomes Quality Initiative definition of chronic kidney disease by stage

Stage	Description	eGFR (ml/min per 1.73 m^2^)
1	Kidney damage with normal or ↑ GFR	≥ 90
2	Kidney damage with mild ↓ GFR	60–89
3	Moderate ↓ GFR	30–59
4	Severe ↓ GFR	15–29
5	Kidney failure	<15 or dialysis

*eGFR* estimated glomerular filtration rate

### Risk factors for developing CKD

Many patients will have renal disease at the time of liver transplant secondary to hepatorenal syndrome and/or acute tubular necrosis. In one study, renal histology pretransplant revealed glomerulosclerosis and other mild glomerular changes with mesangial matrix expansion, capillary-wall changes, and mesangial immunoglobulin (Ig) G, IgA, and IgM in the majority of cases despite normal serum creatinines [21]. Ultrasonographic findings included nephromegaly and increased echogenicity, which improved in the majority of cases after transplant [22]. In pediatric patients, there can be renal involvement from the primary liver disease, as in patients with primary hyperoxaluria, autosomal recessive polycystic kidney disease, Alagille’s syndrome, and tyrosinemia (Table 3). Renal dysfunction in patients with these disorders often improves after liver transplant. However, the long-term affects of preexisting renal disease on developing CKD after orthotopic liver transplant has not been studied systematically in pediatric patients. In contrast, in adult liver transplant patients who develop acute renal failure during the peri- and postoperative periods, there is an increased risk for developing CKD after transplant [23] and progression to CKD stage 5 requiring dialysis [24].

**Table 3:** Liver diseases associated with preexisting renal disease

Tyrosinemia
Autosomal recessive polycystic kidney disease
Alagille’s syndrome
Primary hyperoxaluria
Hepatitis B- and C-related glomerulonephritis

Other identified risk factors (Table 4) for CKD include GFR <70 ml/min per 1.73 m^2^ at 1 year after transplant and cyclosporine use [1]. In this study, there was an inverse relationship between hypertension at 1 year after transplant and development of renal dysfunction. In addition, in adult patients, preexisting diabetes, pre- and postoperative renal failure, hypertension, age, female gender, and hepatitis C infection increased the risk of CKD and ESRD [20]. Though the majority of renal dysfunction after liver transplant is attributed to the use of cyclosporine and/or tacrolimus, there have been reports of other types of pathology present leading to CKD. Studies have found preexisting renal disease such as focal segmental glomerulosclerosis (FSGS) and hepatitis-C-related injury including membranoproliferative glomerulonephritis, unresolved hepatorenal syndrome, and diabetic nephropathy on renal biopsy after liver transplant [25, 26].

**Table 4:** Risk factors for developing chronic kidney disease by type of transplant

**Risk factor common to all transplant types**
Calcineurin inhibitor use
**Liver**
GFR of <70 ml/min per 1.73 m^2^ at 1 year after transplant
Pretransplant renal dysfunction
Acute renal failure
Preexisting diabetes
Age
Female gender
Hepatitis C
**Cardiac**
Pretransplant dialysis
Hypertrophic cardiomyopathy
African American race
Previous transplant
Pretransplant diabetes
Extracorporeal membrane oxygenation use
**Heart–lung**
Hypertension posttransplant
Elevated serum creatinine at 1 month posttransplant
**Hematopoietic stem cell transplant**
Acute graft-versus-host disease grades II–IV
Older age
Transplant from an unrelated donor
Acute renal failure
Chronic graft vs. host disease
Total body irradiation

### Calcineurin inhibitors

The use of cyclosporine (CSA) and tacrolimus in managing liver transplant patients has greatly improved outcomes. However, the improvement in survival is associated with an increased development of CKD. The manifestations of CSA toxicity range from asymptomatic azotemia and proteinuria to fulminant multiorgan failure [27]. In addition to the commonly described striped fibrosis and arterial and vascular lesions seen in patients on these medications, thrombotic microangiopathy has also been described in as many as 50% of patients after liver transplant [25]. These lesions are typically characterized by mesangiolysis, thrombus formation within glomerular capillaries, and widening of the subendothelial spaces. The nephrotoxic effects of CSA correlate with drug serum levels and therapy duration. CSA is also known to cause arteriolar injury, glomerulosclerosis, and interstitial fibrosis, as well as diffuse expansion of the mesangial matrix [28, 29]. The hypothesized mechanisms behind this injury appears to be vasoconstriction secondary to an imbalance between the vasodilatory hormones such as prostaglandin E1 and vasoconstrictive ones such as thromboxane A2 [30]. Increased synthesis of transforming growth factor (TGF)-β1 by calcineurin inhibitors also contributes to the development of CKD in patients after transplant, and genetic polymorphisms in the TGF-β1 gene have been associated with the development of ESRD after cardiac transplant [31].

Reducing calcineurin inhibitor levels while adding mycophenolate mofetil (MMF) in patients with CKD 5 years after liver transplant resulted in renal function improvements as measured by serum creatinine and creatinine clearance after 24 months of combined therapy, with only minor changes noted in immune function in these patients. Decreases in microalbuminuria were also noted[32]. Some authors have suggested that thrice-daily dosing of cyclosporine leads to decreased nephrotoxicity and only mild histopathologic changes in the kidney after 3 years of follow-up [33]. The authors attribute their findings to more constant trough blood levels and lower, less toxic, peak levels as well as the use of a calcium-channel blockers to manage their patients’ hypertension [33]. In a case series, three children who underwent combined liver and kidney transplant were switched to sirolimus because of prolonged renal failure requiring dialysis and acute calcineurin inhibitor toxicity on biopsy. All three demonstrated improved renal function with cessation of dialysis [34]. These studies suggest that decreasing calcineurin inhibitor exposure by adding MMF or eliminating long-term exposure by switching to sirolimus can have beneficial effects on renal function in children after liver transplantation.

## Cardiac transplantation

### Epidemiology

Approximately 350 pediatric cardiac transplants are performed annually in the USA [7]. In a 2006 report from the International Society for Heart and Lung Transplantation, the incidence of renal dysfunction (defined as an abnormal serum creatinine) was 10% at 5 years after cardiac transplant [6]. Sixty-three percent of patients had hypertension. After 8 years of follow-up, the percentage of patient with renal dysfunction did not change, but 2% of patients were on dialysis or had received a renal transplant. In reports from single centers, the incidence of CKD at approximately 10 years after cardiac transplant in pediatric patients ranged from 7% to 54%; however, only a small percentage of these patients progress to ESRD requiring dialysis and/or renal transplantation [7–10]. Hypertension is a more common finding, occurring in approximately 70% of patients after transplant [7–10]. Some centers actually report an improvement in kidney function in the first year following cardiac transplant and stabilization thereafter [9]. In adult studies, there is an increased risk of mortality associated with development of CKD [35, 36].

### Risk factors for development of CKD after cardiac transplant

A recent study of 2,032 pediatric cardiac transplant patients transplanted between 1990 and 1999 identified pretransplant dialysis, hypertrophic cardiomyopathy, African American race, and previous transplant as risk factors for developing CKD (defined in this study as a creatinine >2.5 mg/dl) [37]. Additional risk factors for developing ESRD included pretransplant diabetes and intensive care unit stay or extracorporeal membrane oxygenation [37]. In the adult population, several risk factors for CKD development have been identified and include older age at transplant, pretransplant serum creatinine, preexisting diabetes, abnormal GFR at 1 year after transplant, hypertension after transplant, and cyclosporine immunosuppression within the first 6 months after transplant [12, 35, 36, 38, 39]. Risk factors for progression to ESRD include postoperative development of hypertension and proteinuria of >1 g/24 h [13, 40].

### Calcineurin inhibitors

Though the nephrotoxic effects of calcineurin inhibitors are well known, data in the pediatric cardiac transplant population are conflicting. After 18 months of triple immunosuppression including cyclosporine, patients’ GFR remained stable, and renal biopsy specimens in four patients did not show signs of cyclosporine toxicity [41]. Minor abnormalities in tubular function resulting in hyperuricemia and hyperkalemia were reported. Similarly, in a study of adult cardiac transplant patients treated with cyclosporine for 5 years, GFR (measured by inulin clearance) remained stable at 66 ml/min per 1.73 m^2^, as did tubular function. The only abnormalities noted were hypertension and the presence of microalbuminuria. However, other studies of pediatric cardiac transplant patients found an association between cyclosporine use within the first 2 months after transplant and decreases in GFR years after transplant [42]. High cyclosporine levels (>500 μg/L) in the first 6 months after cardiac transplant were associated with developing ESRD at anytime after cardiac transplant [43]. In a pediatric study of 14 patients with progressive decline in renal function over 2–5 years, inulin clearance declined from 84 ml/min per 1.73 m^2^ at 1 year to 49.8 ml/min per 1.73 m^2^ at 5 years. Biopsies performed on 13 patients revealed chronic tubulointerstitial lesions of grade II associated with acute changes of vacuolization of the proximal tubules. Arteriolar lesions were also present, along with focal glomerular scarring and fibrosis. Lesion extent correlated with calcineurin therapy duration. After reduction in calcineurin inhibitor dose by 50% and change from azathioprine to MMF, a 67% improvement in GFR (77 ml/min per 1.73 m^2^) was noted 1 year after the change. The authors suggest that this improvement may be related to a decrease in preglomerular vasoconstriction [44]. In a study of pediatric patients treated with tacrolimus after transplant, 41% of patients had elevations in serum creatinine of 1–2 mg/dl approximately 2–3 years after transplant [45].

## Heart–lung and lung transplantation

### Epidemiology

The cumulative incidence of CKD, defined as a doubling of serum creatinine, after lung or heart–lung transplantation varies from 34% at 1 year after transplant to 53% by 5 years after transplant [11]. ESRD occurs in 7.3–16% of patients 5 years after transplant [11–13]. The majority of patients, 51%, developed CKD stage 3 by 1 year posttransplant [12].

### Risk factors for CKD after heart–lung and lung transplant

Risk factors identified for developing CKD in a study of 219 patients undergoing a lung or a heart–lung transplant include cumulative periods of a diastolic blood pressure >90 mmHg and serum creatinine value at 1 month posttransplant [11]. In this same study, the authors found that tacrolimus use in the first 6 months posttransplant decreased the risk of CKD compared with those who received cyclosporine alone. Other studies found GFR at 1 month to be a predictor of later CKD [12]. Development of postoperative hypertension is associated with an increased risk of ESRD after lung and heart–lung transplants [13].

## Hematopoietic stem cell transplant

### Epidemiology

The cumulative incidence of CKD varies from 13% to 60% in adult studies [15–17] to as high as 62% in children [14]. CKD usually becomes apparent 6–12 months after HSCT, although it has been described as early as 2 months and as late as 10 years posttransplant. Though this section discusses injury that occurs after HSCT, baseline or pretransplant renal function can impact the results [46]. Therefore, baseline assessments not only of serum creatinine but also urinalyses and more formal estimations of GFR are warranted. Accurate assessment of baseline renal function can help guide later medication dosing.

There are three distinct clinical manifestations of renal disease that can occur in the HSCT patient: thrombotic microangiopathy (TMA), typically hemolytic uremic syndrome (HUS), graft-versus-host-disease (GVHD)-related CKD, and nephrotic syndrome (NS). TMA syndromes represent a spectrum of clinical diseases characterized by systemic or intrarenal platelet aggregation, thrombocytopenia, and microvascular fragmentation of erythrocytes. Platelet aggregation can result in ischemia and organ injury. When renal injury is predominant, a diagnosis of HUS is usually rendered, whereas the presence of extensive extrarenal manifestations leads to a diagnosis of thrombotic thrombocytopenic purpura (TTP) (reviewed in [47]).

### Histopathology of TMA after HSCT

Microscopic examination of kidney biopsy specimens from patients with TMA-associated CKD demonstrates mesangiolysis and loss of endothelial cells with expansion of the subendothelium and occlusion of capillary loops. On electron micrographs, there is extensive widening of the space between the glomerular basement membrane and the subendothelium, with amorphous deposits that are not immune complexes.

### Risk factors for HUS after HSCT

Though no clear relationships have been found to date for the development of TMA after HSCT, a number of risk factors have been examined. In earlier studies, where HUS was the primary diagnosis, risk factors identified were total body irradiation (TBI) [14, 15, 48, 49] and calcineurin inhibitor use [15, 27, 50–54]. However, in more recent studies of TMA, acute graft-versus-host disease (GVHD) grades 2–4, older age, and transplant from an unrelated donor are the primary risk factors identified [55, 56]. Other investigators identified sinusoidal obstruction syndrome, matched unrelated donors or haploidentical donors, and lymphoid malignancy as significant predictors of TMA after HSCT in addition to the above risk factors [17, 57–59]. However, in children who develop HUS after HSCT, the presumptive risk factor in these studies was TBI used as part of the conditioning regimen. For example, Tarbell et al. studied 44 children (aged 3–15 years) with acute lymphocytic leukemia (ALL) or neuroblastoma (NB) who underwent HSCT [14]. Twenty-nine of these patients were alive and in remission 3 months after HSCT and were evaluated in the study. Eleven patients developed increases in blood urea nitrogen (BUN) and creatinine, and ten were anemic and thrombocytopenic with evidence of hemolysis on peripheral blood smear; they also had elevated lactate dehydrogenase (LDH) levels. In every patient except one, the hemolytic process resolved, yet renal insufficiency persisted. The pathologic findings of mesangiolysis with intraglomerular capillary aneurysm formation in conjunction with laboratory abnormalities support the diagnosis of HUS in these children. In another small study, Antignac et al. described seven children referred to their nephrology clinic with CKD approximately 5–10 months after TBI followed by HSCT [48]. All seven children had leukemia, and all received cyclophosphamide alone or with cytosine arabinoside and vepeside, in addition to single-dose TBI as part of their conditioning regimen. Three patients developed CKD without hypertension, and four developed HUS with severe hypertension and microangiopathic hemolytic anemia. Of these, two of the four had normalization of their renal function. Follow-up biopsies, however, showed extensive scarring of the renal parenchyma but almost complete resolution of the mesangiolysis. The glomeruli were globally sclerotic, ischemic, or demonstrated mesangial hypercellularity. Thus, there was evidence of persistent and progressive renal damage in these patients despite normalization of serum creatinine and urinalysis. The occurrence of two different clinical presentations, CKD without hypertension and HUS with hypertension, but similar pathology in these children supports the notion that this is a spectrum of disease rather than distinct pathophysiologic processes.

### GVHD-related CKD

GVHD-related CKD in this patient population is usually defined as an elevated serum creatinine or an abnormal GFR 6–12 months after transplant. The incidence of GVHD-related CKD in children after HSCT varies from 11% to 41% [60–63]. In one recent study, the incidence of CKD (GFR <70 ml/min per 1.73 m^2^) changed over time, with 41% of children having CKD at 1 year, 31% at 3 years, and only 11% 7 years after transplant [63]. In approximately 19% of patients, hematuria and proteinuria persisted up to 10 years after HSCT. Berg and Bolme followed 44 children with acute lymphocytic leukemia (ALL), acute myeloblastic leukemia (AML), and severe aplastic anemia (SAA) and found a significant decrease in GFR 1–2 years after HSCT when compared with their baseline GFR (ALL and AML groups) or with a healthy control group despite serum creatinines that remained within normal limits. An initial decrease in GFR was followed by stabilization up to 5 years posttransplant [64]. This study supports the contention that serum creatinine is not an accurate measure of kidney function. Serum creatinine level and related estimating equations, routinely used clinical measures to estimate kidney function, are dependent on muscle mass and are influenced by age, race, gender, and weight [65, 66]. Patients undergoing HSCT may have large fluctuations in their nutritional status, muscle mass, and weight that will influence GFR based on estimation equations or serum creatinine levels. Proximal tubular dysfunction has also been described in 14–45% of pediatric patients 1–2 years after HSCT, with initial injury to the proximal tubules being nonspecific as reflected by elevated urinary excretion of alpha-1 microglobulin and beta-*N*-acetylglucosaminidase (β-NAG) followed by more specific damage manifested by decreases in phosphate reabsorption [67].

### Risk factors for GVHD-related CKD

The risk factors for GVHD-related CKD in children are similar to those identified in adult studies. Kist-van Holthe et al. also retrospectively identified risk factors for developing both acute and chronic renal insufficiency in a cohort of 142 children undergoing transplant over a period of 5 years in the Netherlands [68]. All children received allogeneic transplants. Ninety-one children received radiation, and 82 of these 91 received TBI. Twenty-five children (18%) had CKD (defined as a GFR <85 ml/min per 1.73 m^2^). These authors found no correlation between radiation dose used and renal insufficiency at 1 year. In a later study from the same group, only acute renal insufficiency predicted the later development of CKD in patients after HSCT [62]. These studies contradict others in the literature that found TBI to be associated with renal injury [60, 61, 69]. However, the doses used here (5–8 Gy in a single fraction) were much lower than described elsewhere. In a study of 92 pediatric HSCT patients by van Why et al., late renal insufficiency developed in 18 of 64 (28%) patients; in half of these patients, the renal disease persisted for 3 months to 3 years [60]. Amphotericin B use, cyclosporine, and TBI were associated with the later development of CKD. In a large retrospective review of 1,635 children and adults, risk factors for developing CKD after HSCT included acute renal failure and acute and chronic GVHD [17]. In this study, TBI was not associated with development of CKD.

### Nephrotic syndrome (NS) after HSCT

Chronic GVHD may manifest itself in the kidney as NS with or without renal insufficiency (reviewed in [70]). Patients usually present with proteinuria, edema, and hypoalbuminemia. The majority of these case reports demonstrate membranous nephropathy (MN) with subepithelial deposits on biopsy; it is postulated that these deposits are antigen/antibody complexes representing GVHD in the kidney. However, cases of minimal-change disease (MCD), which is thought to be a T-cell-mediated process, have also been described [70]. Comparisons between case reports of MN and MCD after HSCT found that MN occurs in 61% of cases compared with 22% of cases having MCD [71]. The majority of reported patients with MN were slightly older males with a history of acute and chronic GVHD. Both MCD and MN occur later after transplant, at 8 and 14 months, respectively, and tend to occur within 1–5 months of GVHD development and/or the tapering of immunosuppression for their chronic GVHD. MN is more difficult to treat, with only 27% of patients reportedly achieving remission compared with 90% of patients with MCD [71]. Others have reported cases of diffuse proliferative glomerulonephritis, anti-nuclear-cytoplasmic-antibody (ANCA)-related glomerulonephritis, focal segmental glomerulosclerosis, and IgA nephropathy [72–76] occurring after HSCT. The development of each of these diseases seems to be associated with chronic GVHD and/or immunosuppression tapering. Treatment with high-dose prednisone and/or reinstitution of calcineurin inhibitors usually results in resolution of NS. Some physicians have used rituximab successfully in patients with NS after HSCT, typically in cases of MN [77].

## Management of CKD after liver, cardiac, lung, and HSCT

Patients who develop CKD after liver, cardiac, lung, and hematopoietic stem cell transplants are at increased risk of mortality [18, 26, 78]. Transplant physicians and nephrologists should work together starting from the time prior to transplant to monitor these patients closely. More accurate measures of kidney function are needed, and baseline and follow-up iohexol or iothalamate studies to measure GFR may be indicated to identify patients with underlying CKD prior to transplant and to allow early identification and intervention in patients with mild changes in GFR posttransplant. New markers to estimate GFR, such as cystatin C, may be more informative in certain patient populations than is serum creatinine. Cystatin C is a cysteine protease inhibitor expressed by all nucleated cells and is freely filtered by the glomerulus. Serum cystatin C correlates well with measured GFR and more accurately measures kidney function than does serum creatinine in the elderly, cancer patients, diabetics, and renal transplant recipients [79–83]. Normal ranges for cystatin C have been validated in children. The normal reference range for children older than 1 year is 0.7–1.38 mg/dl [84]. An equation to estimate GFR based on cystatin C levels in children has also been created: log (GFR) = 1.962 + [1.123*log(1/cystatin C)] [85]. Urinalyses including a microalbumin to creatinine ratio should also be part of the pretransplant workup, and urinalyses should be monitored closely following transplant.

Animal models of HSCT, and specifically of radiation-induced HUS, offer potential interventions for patients with HUS after HSCT. Angiotensin-converting enzyme inhibitors (ACEI) have been used in rodent models of HSCT-related renal injury. The use of captopril or enalapril at the time of TBI in these animals resulted in less azotemia, lower blood pressures, decreased proteinuria, and long-term preservation of renal function [86]. ACEI and angiotensin receptor blockers (ARBs) also help reduce inflammation and inflammatory markers and reduce circulating levels of TGF-β1 in patients after transplant [87–90]. These agents have also been shown to slow CKD progression and decrease proteinuria in patients with renal disease from various causes [91, 92].

**Fig. 1:**
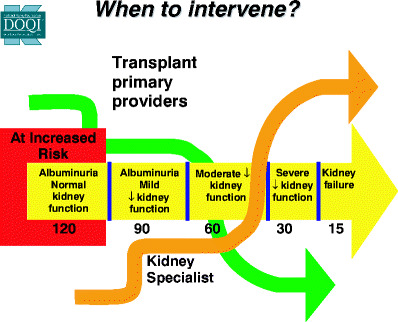
Timing of intervention. *Bold numbers* are estimated glomerular filtration rate based on the Kidney Disease Outcomes Quality Initiative (KDOQI) guidelines. Adapted with permission from [102]

Hypertension management early after transplant is important to prevent CKD development and progression to ESRD in certain transplant populations. ARBs and ACEIs have been shown to be effective and safe in managing hypertension in cardiac transplant patients [93], and ACEIs have been shown to stabilize renal function over 2 years of follow-up [94]. These drugs should be considered as first-line agents to manage hypertension in patients after transplant. In addition, hyperlipidemia management may be important to prevent CKD after transplant. In a study of adult patients undergoing a cardiac transplant, statin use was associated with a decreased risk of development of CKD after transplant [78]. The difficult decision is when to intervene (Fig. 1). Should patients be started on these medications prior to transplant, at the first signs of hypertension and microalbuminuria, or at some set time point after transplant to help protect their kidneys? These decisions need to be made on an individual basis and will vary based on the type of transplant. Frequent discussions between the transplant physician and nephrologist are required to optimize the management of patients with CKD after transplant.

In addition to the above medications, immunosuppression adjustment is important in managing the CKD that develops after transplant, further supporting the need for close collaboration between transplant physicians and nephrologists. In two reported series of lung transplant recipients, sirolimus was used to decrease and eventually stop calcineurin inhibitors in patients with CKD after transplant. Patients had a sustained improvement in kidney function as reflected by a decrease in serum creatinine for 1–12 months [95, 96]. However, prior to changes in immunosuppression, a kidney biopsy may be indicated to define the etiology of the kidney injury and better tailor therapy to prevent or slow disease progression.

Management of patients with ESRD after transplant include the use of peritoneal and/or hemodialysis. Caring for these patients also involves management of complications associated with ESRD, which include anemia, bone disease, hypertension, and metabolic abnormalities. There have been case series of patients undergoing renal transplantation successfully after cardiac, liver or HSCT; renal transplantation is a viable option for patients with ESRD after transplant and can improve outcomes [19, 97–101].

**Questions**

(Answers appear following the reference list)

1. Which of the following are risk factors for developing CKD after cardiac, liver, and HSCT?
  - Calcineurin inhibitors
  - Total body irradiation
  - Acute renal failure
  - Hypertension
2. All of the following are manifestations of CKD seen in patients after HSCT except:
  - Nephrotic syndrome
  - Acute glomerulonephritis
  - Thrombotic microangiopathy
  - GVHD-related CKD
3. What pretransplant factors increase the risk of CKD in pediatric cardiac and liver transplant patients?
  - Preexisting renal disease
  - Race
  - Pretransplant diabetes
  - All of the above
4. Management of pediatric patients who develop CKD after transplant should include all of the following except:
  - Reducing exposure to calcineurin inhibitors
  - ACEI and/or ARB
  - Calcium-channel blockers
  - Statin use
5. What is the most common cause for developing TMA after transplant in patients receiving a heart, liver, or stem cell transplant?
  - Graft-versus-host disease
  - Total body irradiation
  - Calcineurin inhibitor use
  - Diabetes

**Answers:**

1. a and c
2. b
3. d
4. c
5. c
